# Seismic evidence for a 1000 km mantle discontinuity under the Pacific

**DOI:** 10.1038/s41467-023-37067-x

**Published:** 2023-03-27

**Authors:** Zhendong Zhang, Jessica C. E. Irving, Frederik J. Simons, Tariq Alkhalifah

**Affiliations:** 1 Department of Geosciences, Princeton University, Princeton, NJ USA; 2 Department of Earth, Atmospheric and Planetary Sciences, Massachusetts Institute of Technology, Cambridge, MA USA; 3 School of Earth Sciences, University of Bristol, Bristol, UK; 4 Earth Science and Engineering Program, King Abdullah University of Science and Technology, Thuwal, Saudi Arabia

**Keywords:** Seismology, Geophysics

## Abstract

Seismic discontinuities in the mantle are indicators of its thermo-chemical state and offer clues to its dynamics. Ray-based seismic methods, though limited by the approximations made, have mapped mantle transition zone discontinuities in detail, but have yet to offer definitive conclusions on the presence and nature of mid-mantle discontinuities. Here, we show how to use a wave-equation-based imaging method, reverse-time migration of precursors to surface-reflected seismic body waves, to uncover both mantle transition zone and mid-mantle discontinuities, and interpret their physical nature. We observe a thinned mantle transition zone southeast of Hawaii, and a reduction in impedance contrast around 410 km depth in the same area, suggesting a hotter-than-average mantle in the region. Here, we furthermore reveal a 4000–5000 km-wide reflector in new images of the mid mantle below the central Pacific, at 950–1050 km depth. This deep discontinuity exhibits strong topography and generates reflections with polarity opposite to those originating at the 660 km discontinuity, implying an impedance reversal near 1000 km. We link this mid-mantle discontinuity to the upper reaches of deflected mantle plumes upwelling in the region. Reverse-time migration full-waveform imaging is a powerful approach to imaging Earth’s interior, capable of broadening our understanding of its structure and dynamics and shrinking modeling uncertainties.

## Abstract (web summary)

This study uses reverse-time migration full-waveform seismic imaging to reveal a thinned transition zone and a 1000-km mid-mantle discontinuity under the Pacific near Hawaii which they link to the upper boundary of upwelling plume material.

## Introduction

A planet can be characterized by mapping its internal boundaries, the loci of rapid mineralogical change, whether in composition or in phase^1^. Such boundaries generally coincide with first-order contrasts in impedance (the product of mass density and seismic wavespeed) that distort the seismic wavefield and produce observable reflections and conversions of distinct seismic phases. Characterizing seismic discontinuities in the upper- and mid-mantle advances our understanding of the mineralogical and geodynamical state of the mantle^2–4^.

Seismologists have confirmed the global existence of mantle transition zone discontinuities^5–7^ at depths around 410 and 660 km, associated with pressure- and temperature-induced phase transitions in the olivine system^8^. The impedance contrast across the 410 km discontinuity may help constrain the presence of melt, water, and other chemical heterogeneities^9–11^. The 660 km discontinuity separates the transition zone from the mid-mantle and often shows broadened, and complex, reflection signals^12^. Mineral physics shows that at about 410 km depth, olivine transforms to wadsleyite, a reaction marked by a positive Clapeyron slope (*dP*/*dT* > 0) in pressure-temperature space. Wadsleyite gradually transforms to ringwoodite around 520 km depth (*dP*/*dT* > 0), and finally to bridgmanite and magnesiowüstite near 660 km, with a negative Clapeyron slope (*dP*/*dT* < 0). Majorite garnet may be present, complicating interpretation by transforming near 660 km into an Al-bearing perovskite with a positive Clapeyron slope (*dP*/*dT* > 0)^13^. The variable mantle transition zone thickness is interpreted, within the context of mineralogical composition^13,14^, as sensitive to temperature differences from the ambient mantle^8^. Using average mantle properties and published estimates for the relevant Clapeyron slopes^15^, a 100 K temperature increase implies a ~15 km thinning of the mantle transition zone (hereafter: MTZ).

There are also mid-mantle discontinuities observed more intermittently, linked to a range of tectonic environments^16–25^. The stagnation of downgoing slabs, impeded by increases in mantle viscosity, is consistent with mid-mantle discontinuities observed in subduction zones^24,26^, whereas the mechanisms producing mid-mantle discontinuities in areas of mantle upwelling and in tectonically stable regions have not yet been clearly established^19,23–25^. Mid-mantle discontinuities appear as localized structures with strong topography in regional studies of reflected and converted phases, e.g., beneath active subduction zones in Indonesia^27^, South America^28^, and Northeast China^29^, where they have been interpreted as indicative of slab stagnation around 1000 km depth^17,30^. Such an explanation does not account for the existence of mid-mantle discontinuities further away from subduction zones^24^, whether under oceanic^25,31^ or cratonic^32^ lithosphere. Various mid-mantle discontinuities have been linked to the presence of mantle plumes^23,33^. Of particular interest, several such discontinuities have been detected in close proximity to the Hawaiian islands^19,20,24,34^, our area of investigation, where there is an actively upwelling mantle plume^35^. Mid-mantle reflectors seen in receiver-function and transition zone underside reflection studies agree with the depth of a velocity jump observed in tomographic models^23–25^, but whereas tomography shows high wavespeeds overlying low wavespeeds in those areas, receiver functions instead image an impedance that increases with depth.

Tomography models, which use transmitted phases, do not universally agree on the mantle structure below the Hawaiian region^34,36–38^. Transmission tomography is inherently less sensitive to mid-mantle discontinuities caused by either density or velocity change, insufficiently resolving velocity anomalies at those depths^24^. While it may confidently detect the presence of vertically coherent wavespeed perturbations, it is less able to resolve horizontal layering, especially at isolated island stations in the central Pacific, where mantle body-waves arrive at steeply dipping angles^39^.

Seismic imaging methods, including receiver-function analysis, (waveform) tomography, and reverse-time migration, map a variety of seismic data (time-series records of ground motion due to earthquakes) onto a model (an image) of the physical parameters characterizing Earth’s interior. All seismic imaging methods employed today are limited in their ability to reveal Earth’s heterogeneity—they are contingent on the completeness of the physics in the wavefield propagation method, the utilization of specific seismic phases, and data coverage. Waveform tomography primarily resolves smooth perturbations of model parameters with respect to a background model, while seismic migration is designed to capture impedance contrasts, i.e., discontinuities—where seismic reflections and conversions originate^40–42^.

Ray-based imaging methods such as common-conversion-point (CCP) stacking of precursors to surface-reflected body waves, and receiver-function analysis of three-component waveforms assume single scattering within a dominantly horizontally layered Earth^43,44^. In addition, the actual conversion point is unknown for dipping layers and the time-domain image profiles need to be converted to depth sections. In both cases, ray-based methods may easily misplace the imaged structures. Our procedure of reverse-time migration (RTM) uses the reflected wavefield and some of the same data types (precursors to surface reflections including conversions) as those ray-based imaging methods, but we numerically solve the full wave equation instead of contending with the infinite-frequency (ray) approximation to wave propagation. RTM calculates reflector images where the incident waves meet the reflected waves^45^. It involves three steps: (1) forward modeling of the seismic wavefield from known sources through a given velocity model, (2) backpropagating time-reversed seismic reflections using the same velocity model, and (3) applying the imaging condition (a zero-lag cross-correlation of the forward- and backward-propagated wavefields). RTM is capable of imaging complex reflectors embedded in a heterogeneous Earth without human picking of precursory phases. However, RTM relies on densely distributed seismic recording stations for adequate reflector illumination, in order to avoid aliasing, and for sufficient stacking. It also requires large amounts of computational resources and memory storage for solving the wave equation at a global scale. Technical details of our imaging method can be found in *Methods* and Supplementary Information.

In this study, we use three-component seismograms in time windows that may contain *PP*, *SS*, *PS*, and *SP* precursors as our input data, selected based on their predicted travel times in a one-dimensional (1-D) reference Earth model. Wavefields are back-propagated in the transversely isotropic global three-dimensional (3-D) Earth model GLAD-M25^38^ using the spectral-element method, which incorporates ellipticity, self-gravitation, rotation, ocean loading, and attenuation^46^. An impedance-kernel imaging condition^47^ is applied to yield the final interpretable images. We choose to concentrate our imaging effort on the central Pacific region, which is well illuminated by suitable source-receiver geometry.

## Results

### Data

**Fig. 1:**
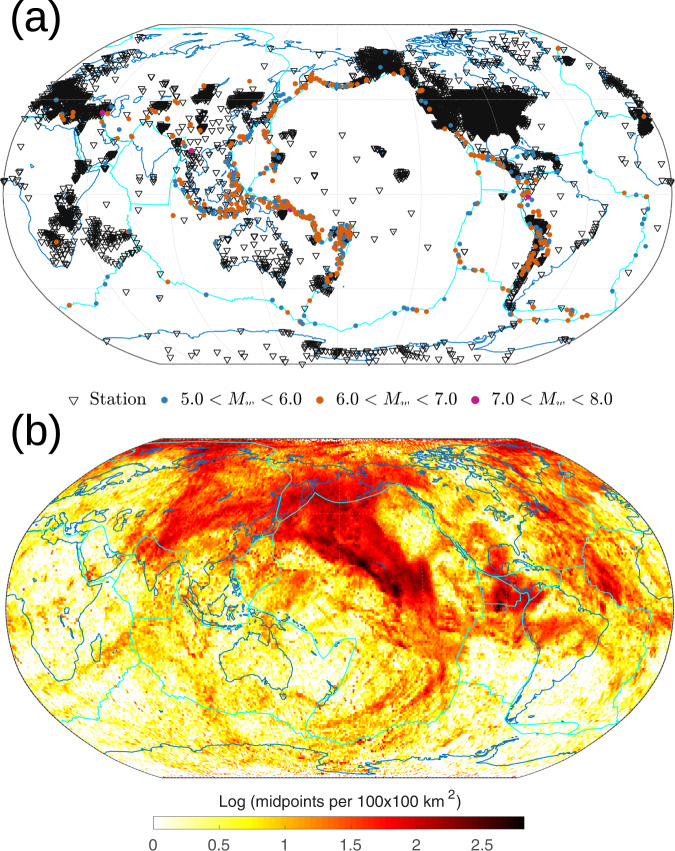
The geometry of earthquake sources and stations used in this study. **a** The distribution of 600 earthquakes (circles color-coded for magnitude) and 8,642 seismic stations (triangles) used in this study, and **b** the corresponding source-receiver *PP* and *SS* midpoint distribution, shown as counts per 100 × 100 km^2^. Not all stations are simultaneously active for all earthquakes. The coverage, while global, is densest in the central Pacific area of interest. Note that we do not display the *PS* and *SP* bounce points, which provide additional illumination. In our images, the dominant contribution is from *SS* precursors.

We selected 600 earthquakes from the global centroid-moment tensor (CMT) catalog^48^ with moment magnitudes ranging from 5.5 to 7.2. The earthquakes were relocated and their focal mechanisms were updated using the GLAD-M25 tomographic Earth model^38,49^. Figure 1a shows a map of the earthquakes selected for study and the in total 8,642 recording stations, resulting in 838,669 station-event pairs with at least one available component. The densely deployed USArray^50^ provides superior illumination of the central Pacific. Figure 1b shows the source-receiver midpoint distribution, using a bin size of 100 × 100 km^2^. The central Pacific has the largest number of midpoint counts, which guarantees sufficient illumination for this region. Our synthetic tests (see Supplementary Information) further confirm the sufficient seismic illumination of the target area. Thus, although the image that we will obtain is defined globally, we focus solely on upper- and mid-mantle discontinuities observed in the central Pacific.

**Fig. 2:**
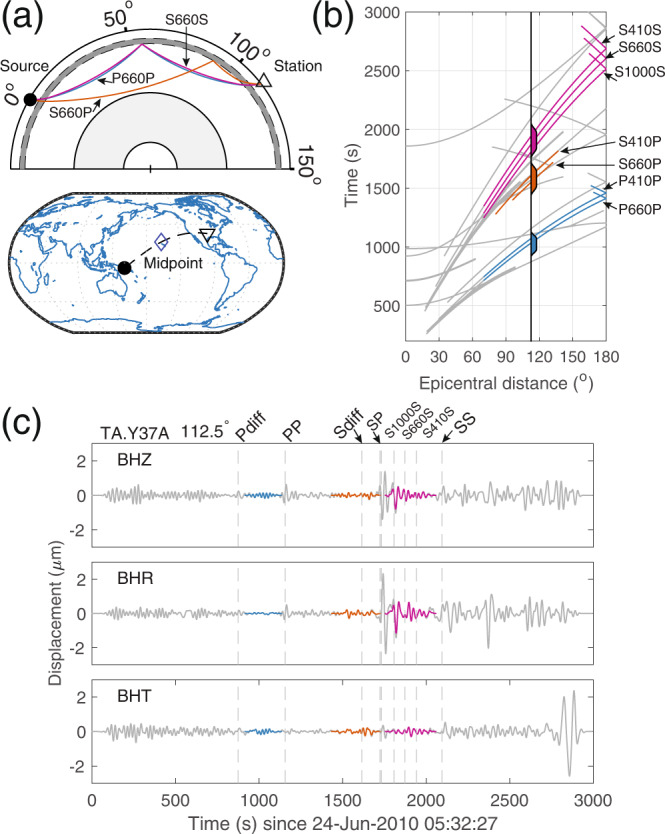
Wave trajectories and time windowing of seismic precursors. Seismic ray paths **a** and travel time-distance curves **b** of the 410 and 660 km-discontinuity (dashed in panel **a**) surface-reflected precursors, calculated in the 1-D reference model PREM. The selection windows, shown in panel **b**, are based on the traveltimes of the target seismic phases. c Example traces (vertical, radial and transverse components denoted BHZ, BHR, and BHT, respectively) used for imaging, for the particular case of seismic event C201006240532A, filtered between 20 and 100 s. Color coding matches between the panels in this figure.

Figure 2a shows typical wavepaths of precursory phases sensitive to the 410 km and the 660 km discontinuities. We defined three selection windows for reflection precursors based on traveltimes in the 1-D reference model PREM^51^, calculated with the TauP Toolkit^52^. We focused on events with epicentral distances 70° ≤ Δ ≤ 180°, where interference from transmitted waves is small [such that precursors are also strong enough for conventional imaging methods,^21^]. Each window is 300 s wide, centered at the predicted arrival time for the 410 and 660 km-discontinuity reflected phase. Cosine tapers were applied to the edges of the time windows. Figure 2b shows travel time curves and the selection windows. In addition to the *PP* and *SS* precursors whose midpoint coverage was rendered in Fig. 1b, *PS* and *SP* conversions may also provide illumination at their bounce points. All data windows contribute to the modeling, but the *SS* precursors dominate in constraining the ultimate image. Figure 2c shows example data for one source-receiver pair after applying the selection windows. The Supplementary Information shows the recovery of MTZ discontinuities in PREM using our methodology.

Waves reflected or scattered off seismic discontinuities are weaker in amplitude than transmitted waves, and often appear at or below the noise level of seismic traces. It is neither practical nor necessary to identify precursors individually within each seismic record. In selecting a wide range of time windows that may contain possible precursors, coherent arrivals are summed constructively by our procedure, provided the background velocity model is sufficiently accurate (see Supplementary Information). Equally, there is no need to stack the data prior to the imaging step. Signal-to-noise enhancement is achieved in the modeling domain instead, by reverse-time migration and the imaging condition.

### Modeling

The USArray data we use are abundant and of high quality, amply sufficient to study the central Pacific region in depth. The technique used in this study, reverse-time migration full-waveform imaging, see *Methods*, is different from waveform tomography using transmitted waves (see Supplementary Information), or ray-based receiver-function imaging based on waves converted underneath seismic stations. Our wavefield extrapolation takes into consideration three-dimensional heterogeneity in the Earth in a manner that is more accurate than could be expected from ray-based approximations. Using an adjoint-state formulation, we back-propagate underside reflections, precursory body-wave phases, sensitive to the bounce point between source and receiver, by solving the elastic wave equation in a realistically complex tomographic background model, GLAD-M25^38^.

Since our imaging procedure relies on a preconditioned adjoint operator to approximate the full inverse solution, the impedance jumps estimated across the imaged reflectors are not absolutely accurate, though they are interpretable in a relative sense^53,54^. We may further approximate the formal inverse (creating what is known as a “true-amplitude" image) by rescaling the amplitudes of the imaged reflectors. With known impedance contrasts of a synthetic Earth model and given the corresponding seismic image, we can estimate such scaling factors for the discontinuities at 410, 660, and 1000 km. The Supplementary Information provides an example of such correction and compares the impedance contrasts of the MTZ and mid-mantle discontinuities near Hawaii. Polarity and phase information in the image are taken into account to track the mapped discontinuities, and the interpretation of our final image will focus on the relative amplitudes of 410, 660 and 1000 km reflectors.

### Mantle discontinuities below the Hawaiian seamount chain

**Fig. 3:**
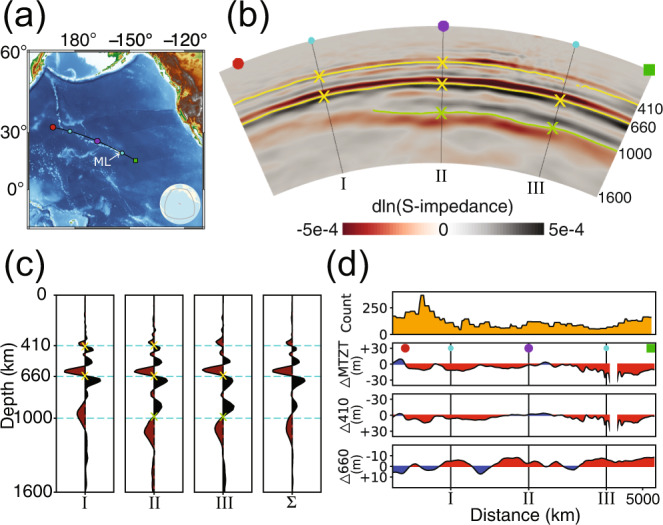
Our reverse time migration image of mantle transition zone and mid-mantle discontinuities along the Hawaiian seamount chain. **a** Geographical situation. **b** A vertical slice through the imaged impedance contrasts. The traced 410, 660, and 100 km discontinuities are drawn in yellow. Three vertical profiles through this section are plotted in **c**. Their stacked profile is in the last panel. **d** Topography of the mantle transition zone discontinuities. The first row shows the midpoint counts extracted from Fig. 1b along the Hawaiian seamount chain.

We focus first on the mantle discontinuities beneath the Hawaiian seamount chain, shown in Fig. 3a. The high midpoint counts (Fig. 1b) along this corridor yield a favorable signal-to-noise ratio in the final image. Figure 3b is the migrated section, obtained as described in *Methods*. We read the location of an impedance discontinuity at the zero crossing between a pair of alternating pulses in the migrated section, which, due to finite-frequency and propagation effects, broaden with depth, and whose polarity and size are indicative of the sign and relative strength of the impedance contrast across the jump. Identifying the zero-crossing as the signature of an impedance jump is verified by inspection of our synthetic images (e.g., Figs. S1 and S3).

Three discontinuities in impedance are recovered in the migrated section: the familiar pair bracketing the MTZ, undulating about 410 and 660 km depth, which are very clearly expressed, and a mid-mantle discontinuity hovering around 1000 km. While the amplitude of the ‘410’ reflector in the image appears to be about a quarter of the ‘660’, the true difference in impedance contrast should be around one-half after amplitude correction. While the previous statement is notionally correct, it is most instructive for the evaluation of our interpretation of the depths and relative amplitudes of the discontinuities to inspect the results from synthetic tests calculated in the 1-D PREM model as can be found in the Supplementary Information. The analysis presented there illustrates the sensitivity of our data to mid-mantle structure, and also shows the results of synthetic tests conducted in the 3-D GLAD-M25 model.

In Fig. 3c, three vertical profiles, labeled I, II, and III, are extracted at different locations, shown in Fig. 3a, from the image shown in Fig. 3b. Their summed stack is labeled Σ in Fig. 3c. The signatures of the reflectors in the image retain the imprint of the correlation between the forward- and backward-propagated wavefields, causing each of them to appear as a wavepacket, i.e., a pair of pulses with opposite polarity, unlike the single impulse expected from simple deconvolution, e.g., with receiver functions. The top panel in Fig. 3d shows the midpoint counts along the imaging line rendered in Fig. 3a. The seismic coverage provides an overall balanced illumination of the target area, hence amplitude differences between imaged reflectors are more likely caused by variations in impedance contrast than by irregularities in the distribution of earthquakes or stations.

As the nominal depth of the discontinuities in our image is based on the impedance-kernel image condition, we marked the zero-crossings in the image with yellow and green lines and crosses in Fig. 3b, and as crosses in Fig. 3c. The frequency content of the data restricts the vertical resolution with which the image and its profiles can be interpreted. At about one-quarter of the dominant wavelength, the relevant scale lengths are about 75 km at 410 km, some 85 km at 660 km, and around 100 km at 1000 km. Any reflector at 520 km^55^ would be unlikely to separably stand out from its neighbors at 410 and 660 km.

### A mid-mantle discontinuity below the Pacific

As shown by Fig. 3b, c, we do find a robust reflector at about 1000 km below the Hawaiian seamount chain. Ray-theoretical studies have indicated the existence of such a reflector in this region^19,20,24^, though in the case of^23^ the polarity of the imaged reflector does not agree with the tomography model. We are now in a position to confirm these early detections and contribute a wideband image of the inferred reflector, which allows us to discuss its likely extent and possible origins. The mid-mantle 1000 km reflector exhibits more topographic undulation than the MTZ discontinuities. Its polarity is opposite to that of the ‘660’, indicating an impedance reversal, with a large impedance overlying a smaller one. The amplitude of the ‘1000’ reflector is about a quarter of the ‘660’ after amplitude correction. Based on its imaged width, which is larger than that of either MTZ discontinuity, the contrast at 1000 km is less sharp, even accounting for the longer wavelengths of the wavefield and the larger inverse reflecting angles that contribute to the image at larger depths in the mantle. The synthetic tests shown in the Supplementary Information indicate that such effects are of secondary importance, allowing us to conclude that the 1000 km discontinuity is indeed comparably more diffuse.

### A thinned mantle transition zone below Hawaii

From the ‘410’ and ‘660’ boundary undulations, we compute the thickness variations of the MTZ by taking their depth difference. The second panel down of Fig. 3d shows the thickness change of the MTZ, in km, compared to a 250 km reference thickness. A significant thinning of the MTZ by ~30 km southeast of Mauna Loa implies a high-temperature anomaly on the order of ~ 200 K. Evidence from petrology and numerical convection modeling also is consistent with the presence of hot mantle upwellings nearby^56,57^. The third and fourth panels of Fig. 3d show the deviation, in km, of the ‘410’ and the ‘660’ from their nominal depths. The lower boundary of the MTZ presents a relatively more subdued topography than its upper boundary. The depth variations of both boundaries are weakly correlated except near Hawaii. At the location of profile III, we furthermore observe a lateral gap in the 410 km reflector, suggesting a reduction of the impedance contrast nearby in the second and third panels of Fig. 3d, and which remained unpicked (no crosses) in Fig. 3b, c. Again, high temperatures are invoked to explain this observation as they may lower the impedance contrast across this boundary^11^.

### Mantle discontinuities below the central Pacific

While MTZ discontinuities have been observed globally, mid-mantle discontinuities have appeared only in regional studies. As was apparent in Fig. 3b, the 1000 km discontinuity below the Hawaiian seamount chain fades out near the northwest end of the chain. We suspect it to be a localized structure, aligned with the direction of the chain. To better understand this behavior and that of all three discontinuities in their geographic context, we next expand our target area to other mantle corridors in the central Pacific.

**Fig. 4:**
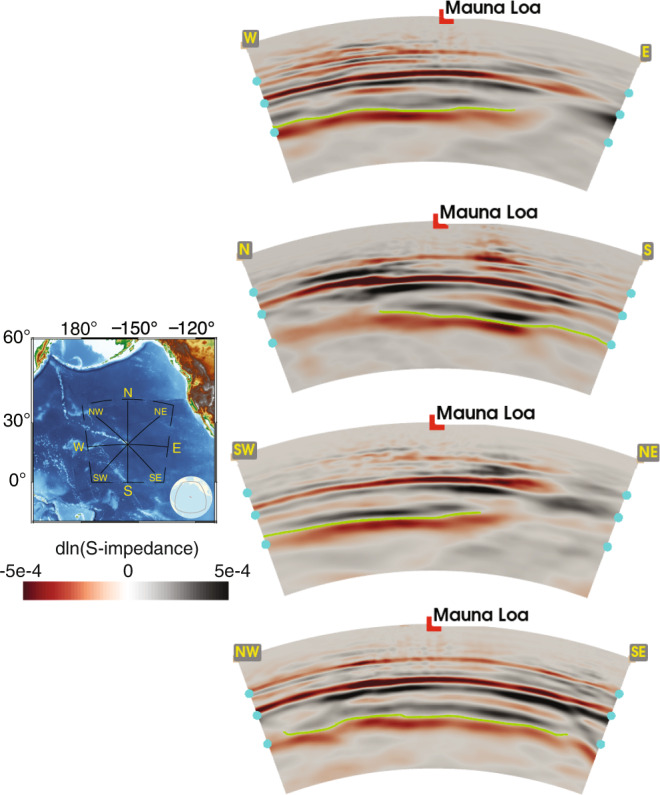
Vertical cross-sections through our impedance image centered at Mauna Loa (see map). From top to bottom: the West-East, North-South, Southwest-Northeast, and Northwest-Southeast sections. The mid-mantle discontinuity inferred from this image is shown by the green lines. See Fig. 3 for the profile aligned with the Hawaiian-Emperor seamount chain.

In Fig. 4 we widen our focus to include a larger imaging area centered on Mauna Loa. Four vertical cross-sections are shown. As already discussed above, overall the 660 km discontinuity is better imaged than the 410 km discontinuity, and the topography of the reflector at 1000 km shows strong lateral variations. Figures 3 and 4 together show how this mid-mantle discontinuity extends along the Hawaiian seamount chain, disappearing gradually towards the northwest, and more abruptly north, northeast, and east of Mauna Loa. We use the image quality of the 660 km reflector in these sections as a proxy to judge the uncertainty of the deeper structures. Our assessment of the imaging quality of the ‘660’ reflector precludes attributing the disappearance of the 1000 km reflector in this area to a lack of data coverage. Synthetic tests shown in Supplementary Information provide a more comprehensive evaluation.

**Fig. 5:**
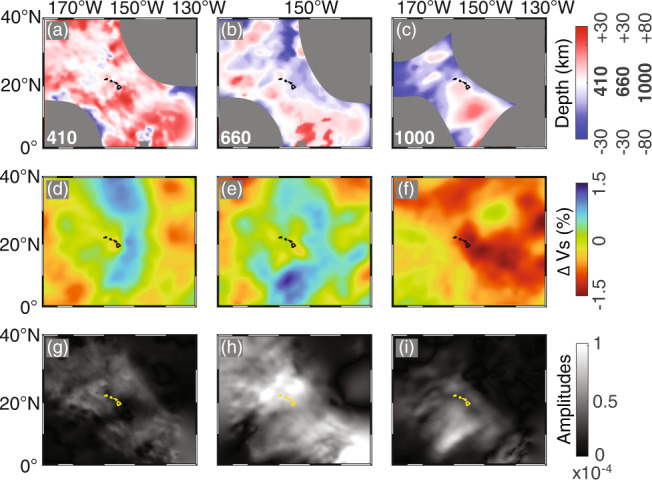
Comparison between discontinuity topography, shear wavespeed anomalies and picked reflector amplitudes. Top row: our imaged topography of the 410 km, the 660 km, and the 1000 km discontinuities. Middle row: depth slices through the GLAD-M25 tomographic model of shear wave speed perturbations at 410, 660, and 1000 km depth. Bottom row: reflector amplitudes extracted from our reverse time migration images.

In Fig. 5 we once again extract the topography and image amplitude of the 410, 660, and 1000 km discontinuities, and show them in map form alongside depth slices of the 3-D velocity model. To ascertain the quality of the structures shown, which remains somewhat variable due to the unevenly distributed source and receiver coverage, we use ray path midpoint counts as a proxy. Areas that are not properly illuminated by this measure are masked. Additional considerations on the relative resolving power of impedance imaging versus velocity tomography are discussed in Supplementary Information.

### Thermal interpretation

The perturbed discontinuities indicate a thinning of the MTZ just below and southeast of Mauna Loa, which is suggestive of a high-temperature anomaly there. Depth slices through the GLAD-M25 model show low shear wavespeeds nearby, which are broadly supportive of that interpretation. Figure 5a, b shows that the upper and lower boundaries of the MTZ below Mauna Loa are located at 425 km and 650 km, respectively. We can also observe depressed impedance contrasts southeast of Mauna Loa, at around 435 km (Fig. 5g), which may indicate local ponding of hot material^58^. A clear deepening of the lower boundary of the MTZ is observed to the south and west of Mauna Loa, see Fig. 5b, consistent with a negative thermal perturbation with respect to ambient mantle.

Typically, a higher than normal mantle temperature is associated with a shallow 660 km discontinuity. However, when mantle temperatures are particularly high, it is thought that the ‘660’ may split or even appear deeper, as the signal from the garnet-perovskite transition may dominate the ringwoodite-bridgmanite transition^14^. The garnet phase transition could enhance upwelling^13^, and it is particularly important in mantle with a higher fraction of basalt^59^. This phenomenon has been observed using receiver functions, for example under Iceland^60^. However, recent receiver function analysis of the mantle under Hawaii did not show such behavior^61^. Our RTM method is not expected to detect a split 660 km discontinuity, as the two different phase transitions are too close in depth to be able to resolve them as separate features using long-period seismic data.

A thermal anomaly outlined by the low shear-wavespeed anomalies in the tomography model, at depths of 410 km (Fig. 5d), 660 km (Fig. 5e), and 1000 km (Fig. 5f) is consistent with the thinning of the MTZ (see also Fig. S8a) and co-located with the deepening of the 1000 km discontinuity (Fig. 5c). The depth variation of the mid-mantle discontinuity, between 950 and 1050 km, is much more substantial than is the case for the MTZ discontinuities, but it is comparable with previous studies^23–25^. Around 1050 km depth southeast of Mauna Loa the discontinuity deepens. The observed polarity change of the 1000 km reflections differs from previous studies^19,23,24^ but is in agreement with at least one tomographic model^37^. The impedance contrasts at 410 km and 1000 km are about 0.48 and 0.23 of those at 660 km under Hawaii, respectively, as interpreted in Supplementary Information.

## Discussion

There is mounting evidence suggesting that mid-mantle discontinuities exist across the globe as local structures^24^. A large number of such discontinuities have been reported in subduction zones, where they can be attributed to the stagnation of subducted slabs^27^. The presence of mid-mantle reflectors has also been reported both in upwelling and tectonically stable regions, but no consensus has emerged as to the underlying physical causes. Shen et al.^19^ observed positive *P*-to-*s* phases converted about 1050 km beneath Hawaii and Iceland, and linked them to a compositional boundary within a silicon-rich lower-mantle body. Using *P*-to-*s* receiver functions, Jenkins et al.^23^ observed reflectors between 975 and 1050 km beneath Western Europe, and hypothesized that chemical heterogeneity within a mantle plume could be their cause. The depth of the reflectors that they imaged coincides with the upper boundary of a low-velocity anomaly revealed by tomographic models. Still, the polarity of the imaged contrast is at odds with the sign of the anomaly. Waszek et al.^24^ sorted the observed mid-mantle discontinuities into upwelling, neutrally buoyant, and downwelling regions, and discovered a preponderance of negative mid-mantle reflectors in upwelling regions compared to elsewhere, suggesting that local mantle heterogeneity is at play. Other regional studies, of seismic discontinuities down to the mid-mantle, with mixed polarities for the reflections off the mid-mantle discontinuities^25,62^, further confound this picture.

No model to date comprehensively explains the observed 1000 km mid-mantle discontinuities. Those imaged using receiver functions coincide with the top of low-velocity anomalies in tomographic models, but are indicative of wavespeed changes that have the opposite sign. In contrast, the negatively polarized reflections from the mid-mantle discontinuity in our images agree well with the velocity changes seen in tomographic models. Although small-scale anomalies such as recycled basalts can also generate negative reflections^23^, a more likely interpretation, supported by tomographic models, is that the mid-mantle discontinuity represents the top of one or more deflected mantle plumes. High-resolution regional tomographic models have hinted at a tree-like structure for some mantle plumes^58,63^. Our imaging results suggest that hot materials originate southeast below Mauna Loa and spread across the central Pacific, extending further along the Hawaiian seamount chain than towards the northern or eastern side of Mauna Loa. Tomography models by Katzman et al.^36^, PRI-S05 by Montelli et al.^35^, and SEMUCB-WM1 by French and Romanowicz^37^ display velocity reversals at this depth. Other tomography models, e.g., S40RTS by Ritsema et al.^64^ or GLAD-M25 by Lei et al.^38^ are not alike in the details of the velocity structure in this region, see the Supplementary Information.

Single-update full-waveform back-propagation-based imaging methods such as ours are well suited for large-scale geophysical problems conducted in realistic settings. Iteratively updating the reflector images based on waveform fitting might further improve the resolution of the reflectors, but carrying out the inversion process is computationally demanding and vulnerable to noise given the small amplitudes of the precursors used. Our procedure is a valuable new approach for structural imaging of upper- and mid-mantle discontinuities with the increasing availability of dense array data and computational resources.

We imaged seismic impedance jumps in the upper and mid-mantle using reverse-time migration full-waveform imaging conducted in a three-dimensional tomographic Earth model, GLAD-M25. The observed data, reflected three-component waveforms, are mapped to upper- and mid-mantle discontinuities. We observed a thinning of the mantle transition zone beneath and southeast of Mauna Loa. A reduction of impedance contrast around 410 km depth is also observed in the same area. We obtained a well-resolved image at depths 950–1050 km below the central Pacific, finding strong evidence for a mid-mantle discontinuity of considerable extent around 1000 km depth. This feature is marked by irregular topography and is indicative of an impedance reversal at this depth. The physical cause for such a discontinuity has not been established yet. One interpretation based both on our high-contrast image and a variety of smooth tomographic wavespeed models is that it may indicate the top of a spreading mantle plume, or system of plumes. In the waveform tomography model used here, GLAD-M25, a low shear wavespeed anomaly coincides with the region where we observe a thinning of the mantle transition zone by about 30 km, though the tomography model itself does not resolve any abrupt velocity changes in the mid-mantle region where we nevertheless clearly pick up a pronounced discontinuity.

## Methods

### Reverse-time migration (RTM) and full-waveform inversion (FWI)

Seismic data recorded at Earth’s surface preserve the time history of seismic waves traveling in the Earth’s interior^65^. Such time history can be reproduced either by backward extrapolation of time-reversed records from a closed recording surface, or via forward extrapolation of the source wavefield within a known Earth model. The model parameters can be estimated from the interaction between the forward- and backward-extrapolated wavefields. Claerbout^45^ formulated the general imaging principle for reverse-time migration (RTM): reflectors exist at space-time points where the downgoing wave meets the waves that travel up or vice versa. Full-waveform inversion (FWI), developed subsequently, uses the wavefield to make sequential model updates distributed around the wavepath^66–68^. Hence RTM has been identified with the first iteration of FWI schemes^69^. Here, we briefly review the details relevant to our study (a flowchart is available in Supplementary Information).

### The adjoint solution

Stated most simply, given a sufficiently accurate Earth model, **m**, seismic data, **d**, can be predicted by a set of equations,

1 $${{{{{{{\bf{d}}}}}}}}={{{{{{{\bf{G}}}}}}}}\cdot {{{{{{{\bf{m}}}}}}}}+{{{{{{{\bf{n}}}}}}}},$$

whereby **G** is a linear(ized) operator, e.g., the wave equation, that relates a model (or model perturbations) to the data, and **n** a noise term. Seeking to match the observed data in a least-squares sense leads to the well-known generalized-inverse solution $\hat{{{{{{{{\bf{m}}}}}}}}}={({{{{{{{{\bf{G}}}}}}}}}^{{{{{{{{\rm{T}}}}}}}}}\cdot {{{{{{{\bf{G}}}}}}}})}^{-1}\cdot {{{{{{{{\bf{G}}}}}}}}}^{{{{{{{{\rm{T}}}}}}}}}\cdot {{{{{{{\bf{d}}}}}}}}$. For typical seismic inversions, the square normal matrix **G**^T^ ⋅ **G** is ill-conditioned, and too large to invert. The often-made approximation **G**^T^ ⋅ **G** ≈ **D**, whereby **D** is diagonal, leads to the “pre-conditioned” adjoint solution

2 $$\hat{{{{{{{{\bf{m}}}}}}}}}\, \approx \, {{{{{{{{\bf{D}}}}}}}}}^{-1}\cdot {{{{{{{{\bf{G}}}}}}}}}^{{{{{{{{\rm{T}}}}}}}}}\cdot {{{{{{{\bf{d}}}}}}}}.$$

From the different types of diagonal approximations to the inverse of the normal matrix studied by Luo^70^, we choose the one that helps compensate for poor illumination, namely

3 $${{{{{{{\bf{D}}}}}}}}\, \approx \int\nolimits_{{{{{{{{\rm{0}}}}}}}}}^{{{{{{{{\rm{T}}}}}}}}}{\partial }_{t}^{2}{{{{{{{{\bf{s}}}}}}}}}^{{{{\dagger}}} }({{{{{{{\bf{x}}}}}}}},T-t)\cdot {\partial }_{t}^{2}{{{{{{{\bf{s}}}}}}}}({{{{{{{\bf{x}}}}}}}},t)\,{{{{{{{\rm{d}}}}}}}}t.$$

Here, **s**^†^ and **s** are the backward- and forward-propagating displacement wavefields, respectively, *T* is the recording interval, **x** and *t* are spatial and temporal variables, and **D** has column and row dimensions equal to those of the model vector **m**.

Known as a pseudo (diagonal) Hessian, **D** compensates for illumination and geometrical spreading of seismic waves, which preserves the relative amplitudes of the imaged reflectors. In practice, we may need additional corrections applied to the “pre-conditioned” RTM image to obtain a “true-amplitude” image. The scaling factors (*α*) can be obtained from the known impedance contrasts ($\hat{{{{{{{{\bf{m}}}}}}}}}$ in Eq. (2)) and the amplitude of their corresponding RTM images (**D**^−1^ ⋅ **G**^T^ ⋅ **d** in Eq. (2)), e.g., Δ*Z*_410_/Δ*Z*_660_ = *αA*_410_/*A*_660_, where Δ*Z* is the impedance contrast of a synthetic Earth model and *A* denotes the amplitude of the corresponding reflector imaged by RTM. With the same source-receiver pairs and a 3-D Earth model that is a good approximation of the long-wavelength of the actual Earth, we assume that the scaling factors learned from the synthetics are also applicable to the real data.

### Wavefield extrapolation

With reverse-time migration, the term **G**^T^ ⋅ **d** in Eq. (1) is firmly rooted in the full physics of wave propagation, unlike ray-based methods. We use the spectral-element solver, SPECFEM_3D_Globe^46^, and a recent high-resolution three-dimensional (3-D) elastic Earth model, GLAD-M25^38^, which is radially anisotropic in the upper mantle. The meshing and simulation parameters are identical to those used by Lei et al.^38^. The effects of attenuation, following the 1-D PREM^51^ model, are accommodated in forward and adjoint simulations. Specifically, we performed our simulations at the scale of the globe, with a minimum resolvable period of 17 s, and for record lengths 50 minutes in duration. A (quasi) Heaviside source time function is used to generate the forward-propagating wavefield. Simulations were performed on the Shaheen II supercomputer at King Abdullah University of Science & Technology (KAUST). One forward simulation takes about 75 minutes, and the calculation of the full gradient about four hours.

### The impedance-kernel imaging condition

The imaging condition combines the forward- and backward-propagated wavefields in a manner that takes the physical nature of scattering and reflection into consideration. Hence, the physical properties of discontinuities can be inferred from a direct interpretation of the obtained image. This study uses the impedance-kernel imaging condition, which focuses on seismic discontinuities caused by abrupt impedance changes^47^. With this imaging condition scattering from large angles is effectively reduced, yielding a high-resolution image, as well as reducing edge artifacts caused by the limited recording aperture.

The shear impedance kernel, ${{{{{{{{\bf{K}}}}}}}}}_{{\rho }^{{\prime} }}$, where ${\rho }^{{\prime} }=\beta \rho$, the product of mass density *ρ* and seismic shear wavespeed *β*, is given by Tromp et al.^66^ and Zhu et al.^47^ as

4 $${{{{{{{{\bf{K}}}}}}}}}_{{\rho }^{{\prime} }}=-\int\nolimits_{0}^{T}\rho ({{{{{{{\bf{x}}}}}}}})\,{{{{{{{{\bf{s}}}}}}}}}^{{{{\dagger}}} }({{{{{{{\bf{x}}}}}}}},T-t)\cdot {\partial }_{t}^{2}{{{{{{{\bf{s}}}}}}}}({{{{{{{\bf{x}}}}}}}},t)\,{{{{{{{\rm{d}}}}}}}}t\\ -\int\nolimits_{0}^{T}{\epsilon }_{jk}^{{{{\dagger}}} }({{{{{{{\bf{x}}}}}}}},T-t)\,{C}_{jklm}\,{\epsilon }_{lm}({{{{{{{\bf{x}}}}}}}},t)\,{{{{{{{\rm{d}}}}}}}}t.$$

Again, **s**^†^ and **s** are the adjoint and forward displacement wavefields, and ${{{{{{{{\boldsymbol{\epsilon }}}}}}}}}_{jk}^{{{{\dagger}}} }$ and **ϵ**_*lm*_ are elements of the adjoint and forward infinitesimal strain tensors, whereas *C*_*jklm*_ are the elastic parameters.

The influence of imaging artifacts can be suppressed by adding data to the modeling. Inasmuch as artifacts may persist in the final image, they can often be exposed as geodynamically implausible structures, hence ignored^41^.

## Supplementary information

Supplementary information

## Data Availability

We acknowledge IRIS (iris.edu) and ORFEUS (orfeus-eu.org) for providing the data used in this study. These data are available from data centers run by IRIS, GEONET, IPGP, ORFEUS, INGV, and ETH. The combination of global (II: 10.7914/SN/II, IU: 10.7914/SN/IU, IC: 10.7914/SN/IC, US: 10.7914/SN/US, CU: 10.7914/SN/CU, GT: 10.7914/SN/GT, GE: 10.14470/TR560404, and G: 10.18715/GEOSCOPE.G), regional (AF: 10.7914/SN/AF, CN: 10.7914/SN/CN, AU, AI: 10.7914/SN/AI, NZ, MN: 10.13127/SD/fBBBtDtd6q, BL, C and JP), and temporary networks (TA: 10.7914/SN/TA) greatly improved the global coverage.

## Code availability

The open-source spectral-element software package SPECFEM_3D_GLOBE used for the numerical simulations in this article is freely available via the Computational Infrastructure for Geodynamics (CIG; geodynamics.org). We used the open-source packages SphModel^71^ and SubMachine^72^ to prepare the grids of tomography models. Computer code and processed data to aid in reproducing the figures in this paper are available at https://github.com/zhen-dong-zhang/Zhang_NCOMMS-2023.
