# Supplementary material for: Seismic evidence for a 1000 km mantle discontinuity under the Pacific
Source: Nat Commun. 2023 Mar 27;14:1714. doi: 10.1038/s41467-023-37067-x (PMC10042893; doi:10.1038/s41467-023-37067-x)
Supplement: Supplementary file 1 — Supplementary information [file 41467_2023_37067_MOESM1_ESM.pdf]

Supplementary Information: Seismic evidence for a 1000 km mantle discontinuity under the Pacific

Zhendong Zhang[1,4], Jessica C.E. Irving[2], Frederik J. Simons[1]
and Tariq Alkhalifah[3]

[1] Geosciences Department, Princeton University, Princeton 08540, NJ.
[2] School of Earth Sciences, University of Bristol, Bristol, BS8 1RJ, UK.
[3] Earth Science and Engineering Program, King Abdullah University of Science and Technology, Thuwal, 23955–6900, Saudi Arabia.
[4*] Department of Earth, Atmospheric and Planetary Sciences, Massachusetts Institute of Technology, Cambridge 02139, MA, USA.

Supplementary Notes

In this supplementary text we provide a brief discussion on seismic impedance modeling and a flowchart of how we obtain our RTM image, to help reproduce our results. We detail synthetic tests, present additional clarification, and provide comparisons with published tomography models to help evaluate our mantle-discontinuity images. For a fair comparison, the imaging process for all tests is identical to that used in our real data application. The only difference is that we replace the recorded waveform data with synthetics. We start with the isotropic version of the one-dimensional (1-D) PREM [1] model, focusing on the basics of seismic imaging and the more advanced RTM imaging of MTZ discontinuities. Data sensitivity analysis indicates that only precursors to surface-reflected seismic phases (*PP*, *SS*, *PS* and *SP*) are sensitive to mid-mantle discontinuities in the Hawaiian region. Next we use the three-dimensional (3-D) anisotropic GLAD-M25 model, upon which we impose shear impedance anomaly to examine its recovery through RTM. We also show the resolution difference between waveform tomography and RTM imaging using the data difference (predictions minus observations, without selection windows) as the input. Finally, we calculate the discontinuity attributes and compare our RTM images with three published tomography models that are widely used for global comparisons [2].

Supplementary Discussion

Impedance, reflectivity, and source signature We start with the very basics of seismic impedance modeling [3]. Fig. S1(a) shows the shear impedance (Z, the product of density and shear wavespeed) of the isotropic PREM model. Seismic discontinuities are defined by abrupt changes in (shear) impedance, and can be characterized by the reflection coefficients, shown for the MTZ in Fig. S1(b). The reflection coefficient in the 1-D case under normal incidence is $c = (Z_2 - Z_1)/(Z_2 + Z_1)$, where Z is labeled with the layers that define the contrast. Seismic reflection data, $d(t)$, are in essence the convolution $d(t) = c * R(t)$, where $R(t)$ is the source wavelet [3]. Estimating the reflection coefficient, c, requires deconvolution of the source wavelet from the data, e.g, by spectral division, $c = \mathcal{F}^{-1}\{\mathcal{F}[d(t)]/\mathcal{F}[R(t)]\}$, where $\mathcal{F}$ denotes Fourier transformation. In practice, the source wavelet is incompletely characterized, and the deconvolution may be unstable.

To remediate this situation we can use the correlation between the forward- and backward-propagated wave fields to approximate the formal inverse solution, which yields the so-called seismic "image". In that case, the adjoint approximation (Eq. 2 in the *Main Text*), retains the imprint of the source wavelet and thus the reflector image takes the form of a pair of pulses with opposite polarity, as shown in Fig. S1(c).

Source-wavelet deconvolution, in theory, can remove these imprints. Yet we did not implement any such a procedure here for two reasons. First, the source wavelet will vary between different earthquakes, and second, deconvolution is vulnerable to

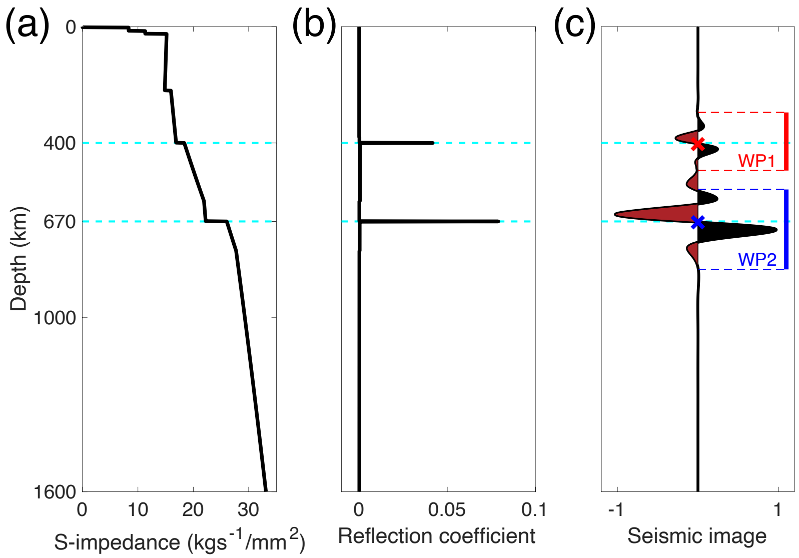

Fig. S1 The finite-frequency signature of seismic reflectors in a 1-D Earth model. (a) The shear (*S*) impedance and (b) MTZ reflectivity of the isotropic PREM model, and (c) how both MTZ reflectors appear under the convolutional model. The red (*WP1*) and blue (*WP2*) segments in (c) are the wave packet responses to the 400 and 670 km discontinuities. Crosses mark what we would estimate to be the locations of the model discontinuities.

division by zero. In the 3-D setting of our study, the shape of the reflector image will be determined by the incident angle, the dominant frequency of the wavelet, and the speed of seismic waves. A maximum resolution of one quarter the dominant wavelength in the vertical direction can be achieved if normal-incidence reflections are available [4]. The horizontal resolution is related to the Fresnel zone and, considering the long wave paths involved here, it is much poorer than the vertical one.

Fig. S2 shows how we obtain the RTM image, to help reproduce our results.

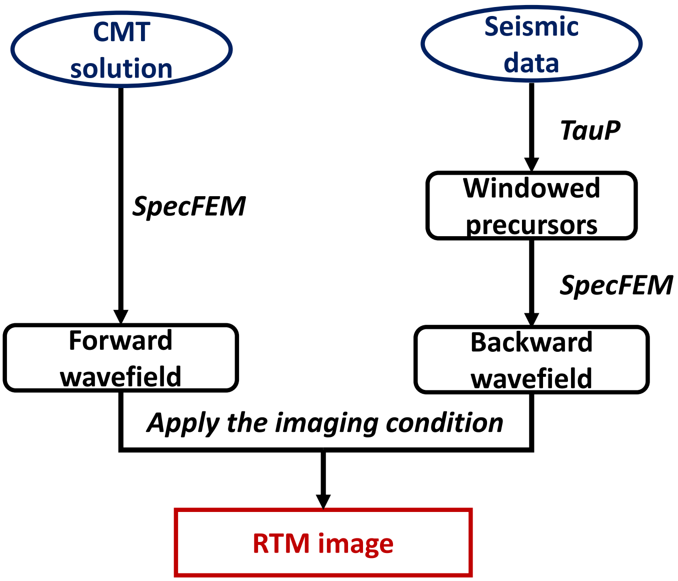

Fig. S2 Workflow for calculating an RTM image. Input data are seismic moment tensors and observed seismic waveforms. We generate the time-selection windows for *PP*, *PS*, *SP* and *SS* precursors based on their traveltime predicted in a 1-D Earth model using the `TauP` package. We then calculate the forward- and backward-propagated seismic wavefields using `SPECFEM_3D_Globe` and apply the impedance-kernel imaging condition. The final RTM image is obtained by stacking the imaging results.

Imaging MTZ discontinuities in PREM In order to verify the recovery of MTZ discontinuities, and to demonstrate that the mid-mantle discontinuity that we imaged in the Pacific does not arise as an artifact of the methodology, we conduct synthetic inversion tests. We simulate synthetic waveforms within the isotropic PREM model via spectral-element modeling, and conduct RTM imaging on the resulting data using the same imaging methodology and with the same data acquisition configuration and window selection as the model presented in the *Main Text*.

The synthetic test results in a clear image of the 670 km discontinuity (Fig. S3a). The discontinuity around 400 km is present but weak and hence not well rendered in the image. We did not select precursor windows related to the 220 km discontinuity within PREM, hence we also do not expect to image a reflector at that depth. Importantly, we do not find any evidence for a mid-mantle discontinuity. Note that

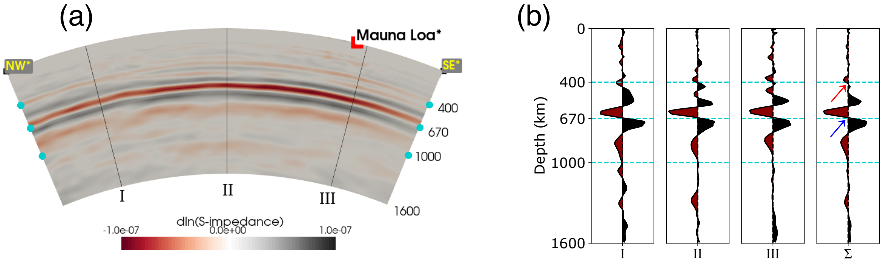

Fig. S3 Synthetic test to evaluate the imaging of MTZ discontinuities within the isotropic PREM model. (a) Image obtained using the geometry of the Hawaiian seamount chain, by simulating waveforms that replicate our data set and modeling them exactly as in the *Main Text*. (b) Three profiles, and their stack, Σ, where the red and blue arrows point to the zero-crossings interpreted as the location of the reflectors. No reflector is imaged at 220 km since our time windows did not select any related data. In this experimental setting the 670 km discontinuity is much stronger than that at 410 km. The RTM image is not suggestive of a mid-mantle discontinuity at 1000 km. Compare with Fig. 3 in the *Main Text*.

we do not interpret the sidelobes at depths around 800 km as signal. Generally, one should not interpret individual negative or positive (red or black) streaks as genuine impedance contrasts.

To better compare the imaged 400 and 670 reflectors, we extract three vertical profiles from the vertical section (Fig. S3b). The stacked profile (last panel) shows clearly imaged reflectors associated with the 400 and 670 discontinuities. We pick the zero-crossings, marked by the arrows, as the reflector depths. The relative magnitude of the reflection coefficients can be roughly estimated from the amplitudes of the reflector signatures in these profiles. The imaged 400 km reflection is about a quarter the size of that of the 670 km reflection. Such a value is close to what we see in the images obtained from the real data in the actual Earth (see Fig. 3c in the *Main Text*), and by comparison with the true model values in Fig. S1(b), this test helps us understand how to interpret the amplitude scalings involved with mapping MTZ discontinuities.

Data sensitivity to mid-mantle discontinuity The likely recovery of model structure from observed data can be assessed by evaluating the sensitivity to those structures of any measurements made. If certain model features fail to generate any expression in the data, real structures will remain unresolved, and spurious structures may arise as artifacts due to nonuniqueness. Different data types will be sensitive to different aspects of model structure. Transmission data, for example, are broadly sensitive to velocity changes along the wave path. Hence the objective of waveform tomography, to update model wavespeeds by minimizing the difference between observed and calculated waveforms of transmitted phases. Reflection data, on the other hand, are most sensitive to impedance jumps between layer boundaries. Hence reflections outperform transmission data in imaging internal discontinuities. Precursor data are often ignored in waveform tomography due to their low signal-to-noise ratio [e.g., see Fig. 2 in 5]. In particular, sharp RTM images are preferred to smooth tomographic images to study mantle discontinuities.

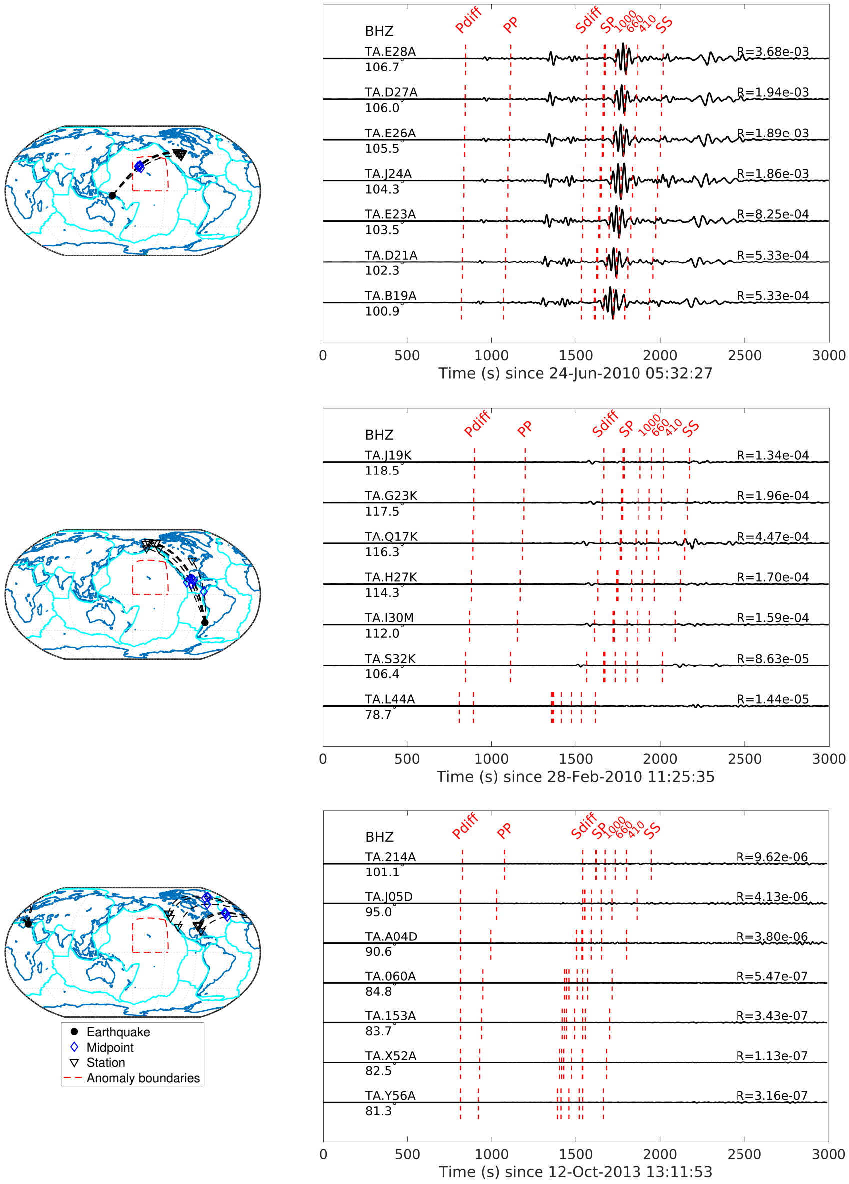

Fig. S4 Waveform difference between synthetics calculated in tomographic Earth model GLAD-M25, with and without a superimposed shear impedance anomaly around 1000 km depth, for different source and receiver pairs. All waveform differences are plotted on the same scale. They are significant only when the bounce points of source-receiver pairs lie within the anomaly boundaries, shown by the dashed red lines. Such differences arise mainly from precursors to the surface-related phases, identifiable from their arrival times.

To confirm the power of imaging mid-mantle discontinuities using precursor data as input for RTM imaging, in Fig. S4, we assess data sensitivity by calculating synthetic waveform differences caused by a shear impedance anomaly inserted around 1000 km depth (as shown later, in Fig. S5a) for three earthquakes, C201002281125A, C201006240532A and C201310121311A.

We quantify the waveform difference by calculating the relevant energy ratio, $R = \langle d^p - d^o, d^p - d^o \rangle / \langle d^o, d^o \rangle$, for each trace, where the angle brackets denote the inner product over the time domain of interest. The three earthquakes chosen are similar in magnitude, depth, and distance to the USArray Transportable Array stations, but they are being observed at different backazimuths. Only one of those earthquakes, C201006240532A, will lead to scatterers that can be imaged below Hawaii when recorded by the array.

Fig. S4 shows their source and receiver pairs, and the corresponding waveform difference calculated in model GLAD-M25, both with and without the synthetic mid-mantle anomaly. The difference waveforms for event C201006240532A contain mainly precursors to surface-related seismic phases, confirming that they are sensitive to mid-mantle discontinuities at certain locations. There are no significant waveform differences if the midpoints of source and receiver pairs lie away from the added anomaly (as is the case for events C201002281125A and C201310121311A) except for one source -receiver combination (C201002281125A and TA.Q17K). In that particular case the wavefield, judging from the ray paths, comes closest to the edge of the anomaly, where boundary scattering or finite-frequency effects may contribute to the waveform difference—in a minor way.

In the absence of mid-mantle discontinuities near their conversion points, waveforms in the precursor time windows show no difference, which validates our inversion approach. We add that non-precursory, direct phases such as *P* and *S*, or unconverted reflections such as *PP* and *SS* are not sensitive to mid-mantle discontinuities below the oceans.

Imaging mid-mantle discontinuities in 3-D To assess the recovery of a sharp impedance contrast imposed upon a smooth tomographic background model, we create a synthetic shear-impedance contrast inspired by the RTM image from the real data (Fig. 3 in the *Main Text*), a regional mid-mantle "layer" that is 100 km thick, between 900 km–1000 km depth (dashed red lines in Fig. S4). The anomaly represents a 10% increase in shear impedance, which yields a reflection coefficient of 0.05, comparable to the reflection coefficients of the MTZ discontinuities within PREM.

Fig. S5(a) shows a vertical cross-section through this synthetic shear-impedance model, along the Hawaiian seamount chain. We next calculate two different RTM images: one within the very same model that was used to generate the synthetics (that is, GLAD-M25 *plus* the artificial anomaly) shown in Fig. S5(b), and another within the same model but without the anomaly (i.e., GLAD-M25), shown in Fig. S5(c). Three vertical profiles (Fig. S5d) are also included for a better comparison. Note that these have discontinuities at 410 km and 650 km, which GLAD-M25 inherited from its starting model, in addition to the velocity perturbation introduced for this test.

Both of the resulting RTM images are close to one other. In both, the MTZ discontinuities and the added shear impedance perturbation are imaged at their actual

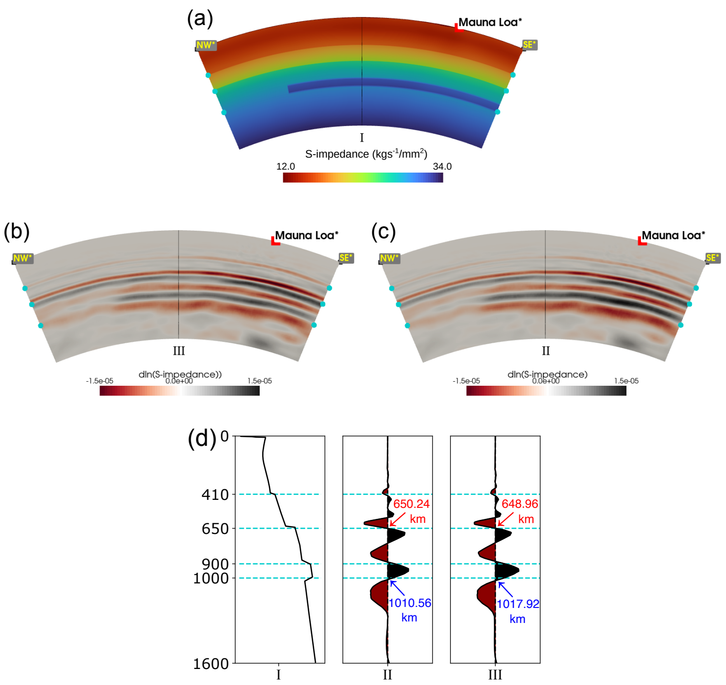

Fig. S5 Synthetic test within a 3-D Earth model. (a) The shear impedance of a synthetic model, GLAD-M25 with a regional perturbation superimposed at 1000 km depth. Using simulated data with all the same characteristics as the real data, and conducting the modeling in exactly the same fashion, we show how the model features are imaged along the Hawaiian seamount chain. (b) Image obtained from RTM using the model shown in (a) as a background velocity model. (c) Image obtained from RTM using the original smooth GLAD-M25 model as a background. (d) Three vertical profiles, one through the impedance model and two through the images, where the red and blue arrows point to the zero-crossings interpreted as the location of the reflectors. The close correspondence of these images confirms that both the globally existing MTZ discontinuities and local anomalies can be imaged at their actual locations, with little effect from inaccuracies in the background velocity model.

locations. The extracted depths of the 650 and 1000 km reflectors computed in the "true" synthetic model are closer to what they should be. We observed an unusually large sidelobe (at about 800–900 km depth) associated with the 650 km reflector (compare with Fig. S3b), which may indicate the upper boundary of the added anomaly. However, due to the low-frequency content of the data used for imaging, the wavelets of these imaged reflectors are not well separated. The relative amplitudes of the 650 and 1000 km reflectors computed in the "true" synthetic model are also more accurate under perfect knowledge. However, the difference with the relative amplitudes in the image calculated within the "smooth" synthetic model is minor. We ignore differences of that order in our interpretation, considering that many other factors affect imaged impedance amplitudes. Indeed, since we know the relative impedance contrasts of the mantle discontinuities in the synthetic Earth model and their image amplitudes from the RTM image, we can calculate the correction coefficients (see *Methods*) and rescale the imaged reflectors for "true-amplitude" imaging.

In our case, we need to double the amplitude of the 410 km reflector and halve the amplitude of the 1000 km reflector of our RTM images to make a more accurate comparison with the 600 km reflector. A more precise correction of the amplitude would require taking into account the lateral position of the image profile and computing the expensive Hessian.

This experiment confirms that RTM imaging is relatively insensitive to inaccuracies in the background model. In reality, we of course do not have access to an “exact” Earth model to calculate the RTM images, but this test confirms that contemporary tomographic models are accurate enough to conduct RTM imaging.

Fig. S6 shows four more cross-sections of the synthetic shear impedance and the corresponding RTM images centered on Mauna Loa. All of these used the unadulterated GLAD-M25 model as background—mimicking the real case where the velocity

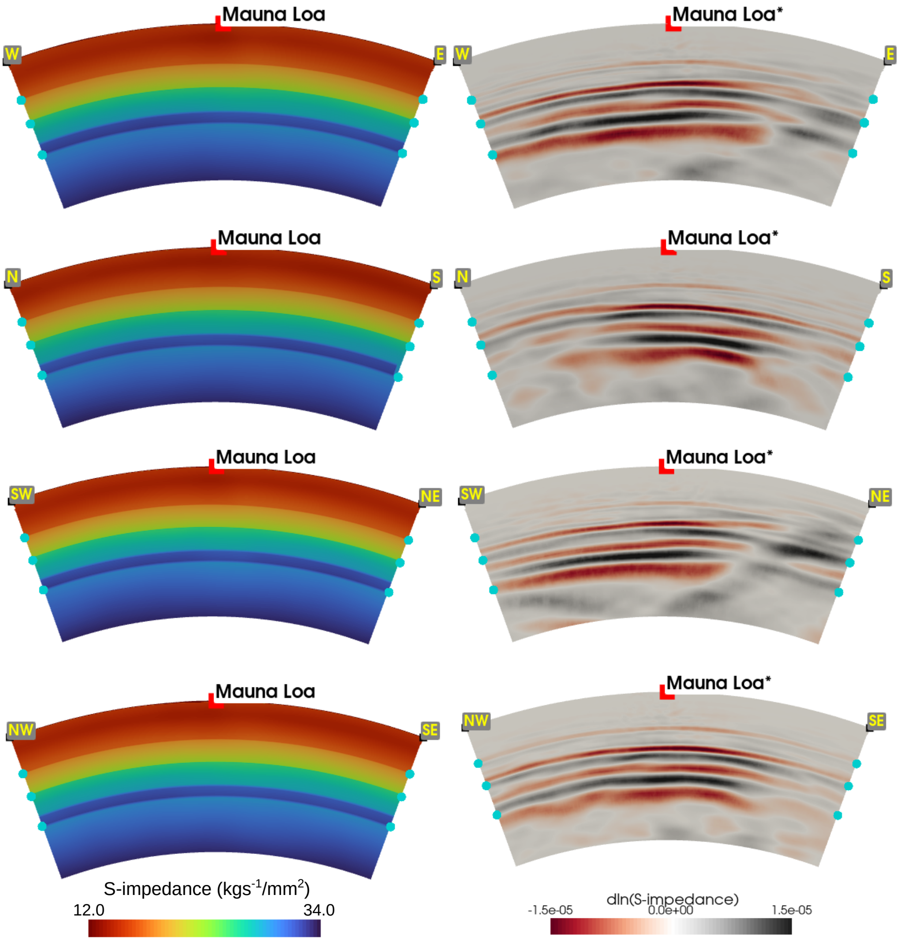

Fig. S6 Vertical cross-sections through the synthetic shear impedance model and the RTM images made on synthetic data migrated within the smooth background model, centered at Mauna Loa (see map in Fig. 4 of the *Main Text*). The image quality is influenced by uneven illumination.

model is smooth, but the imaged discontinuities sharp. As discussed with Fig. 1(b) in the *Main Text*, imaging quality highly depends on the bounce-point density of seismic waves. The northwest-southeast section (i.e., along the Hawaiian seamount chain) is most densely sampled by seismic inverse scattering and thus displays the best imaging quality.

Altogether, these synthetic experiments help evaluate the RTM images that we calculated from the real data, shown in Figs 3(b) and 4 in the *Main Text*. In particular, we interpret the limited extent of the reflectors imaged around 1000 km depth (Fig. 3b in the *Main Text*) as a real and well resolved feature. In contrast, the quality of the imaged reflectors shown in Fig. 4 in the *Main Text* continues to be negatively impacted by poor illumination in the area.

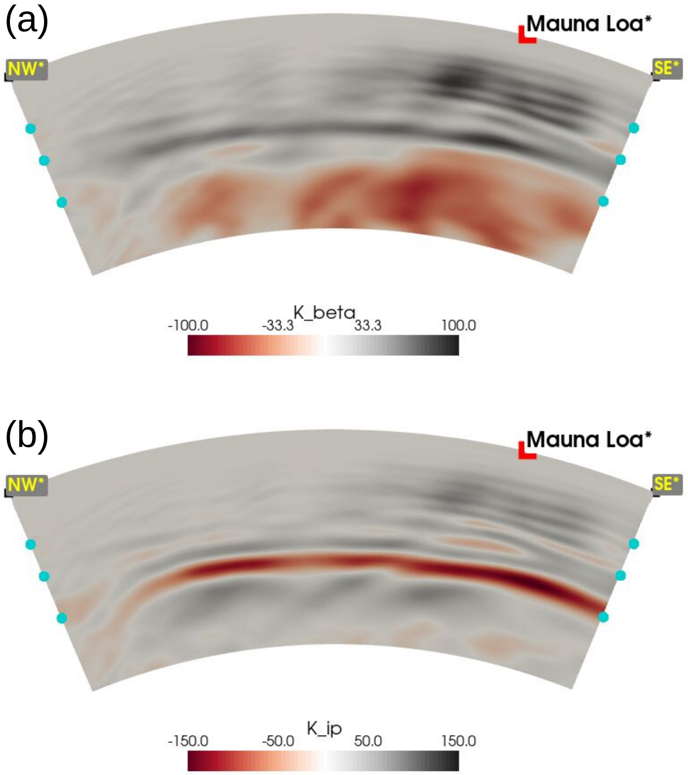

Fig. S7 The difference between waveform tomography and RTM imaging through the lenses of their kernels. Synthetic data are produced within the model of Fig. S5(a) (GLAD-M25 with the layered input anomaly), and synthetic predictions made within GLAD-M25 (without the anomaly). The entire difference between waveforms, without time windowing, is used to construct the shear wavespeed kernel (a) for the first iteration in waveform tomography, and (b) the shear impedance kernel for the RTM imaging step, both shown in cross-section along the Hawaiian seamount chain. Waveform tomography initially recovers smooth wavespeed variations, while migration immediately focuses on sharper contrasts. In both cases imaging imperfections can be alleviated by data selection, and/or applying updates iteratively.

Tomography kernels vs RTM images Waveform tomography and RTM imaging capture different scale lengths of properties within the Earth. As explained in the *Main Text*, RTM imaging can be regarded as a first iteration of full-waveform tomography. However, since both methods use very different types of seismic waves, their sensitivities to Earth's interior are markedly different. Waveform tomography relies on the transmitted wavefield, generating smooth, long-wavelength updates along the wave path. In contrast, RTM migrates reflected waves back to their reflection locations, which yields sharp, high-resolution images of discontinuities. The resolution difference arises from the kernels that are used to perform the model updates.

Here we show two kernels representative for waveform tomography and RTM imaging, respectively, to demonstrate that difference. To generate synthetic data, d^o, we used the perturbed shear impedance model of Fig. S5(a). The GLAD-M25 model is used as the starting model to generate data predictions, d^p, akin to a first iteration in waveform tomography. The data difference, $d^p - d^o$, corresponding to the adjoint source in waveform tomography, was used as input applying any time windowing.

Fig. S7 shows the shear wavespeed kernel and the shear impedance kernel, respectively. Both of these recover the anomaly around 1000 km depth to some extent. The shear wavespeed kernel is smooth with long-wavelength features, and the data residuals are projected across the whole wave path, smeared out and not limited to the location of anomaly. In contrast, the shear impedance kernel exhibits short-wavelength features, and the imaging focuses on the actual location of the anomaly. Imperfections and distortions of the image, e.g., at the edge of the anomaly, are caused by insufficient illumination. The imaging artifacts that appear at MTZ discontinuities are caused by instances of multiple scattering, and can be partially suppressed using judicious data selection windows.

Reflection magnitudes and MTZ thickness We calculate the MTZ thickness and the relative reflector magnitudes of the discontinuities using the results shown in Fig. 5 in the *Main Text*. Figs. S8(a–b) show direct evidence of the thinning of the MTZ and the depressing of the impedance contrast at about 410 km below and southeast of Mauna Loa. An enlarged impedance contrast at about 1000 km southwest of Mauna Loa may indicate the ponding of mantle plumes beneath (Fig. S8c). We stack the profiles of the RTM image close to Hawaii ($20\pm2.5°$N and $-155\pm2.5°$W). Figs. S8(d–f) are the stacked image profiles of 410, 660 and 1000 km reflectors, respectively. We pick the zero-crossing of wavelets as the depth of the discontinuity. The mean value of the peak-to-trough amplitudes is chosen as the reflection magnitude of the reflector. The amplitudes extracted from an RTM image need to be rescaled. From the synthetic tests in Figs. S1 and S3, we learn that the amplitude ratio of the 410 and 660 reflectors may be underestimated by 50%. That of the 1000 and 660 reflectors may be overestimated by a factor of two according to the synthetic test shown in Fig. S6(d) (the strength of the added 1000 km discontinuity is about half that of the 660 km discontinuity but the amplitudes of the imaged reflectors are similar). After rescaling the amplitudes of the 410 km (multiplied by 2) and 1000 km (divided by 2) reflectors (see *Methods* for details), we can estimate the relative impedance contrasts at 410 km and 1000 km to be about 0.48 and 0.23 of the impedance contrast at 660 km, respectively.

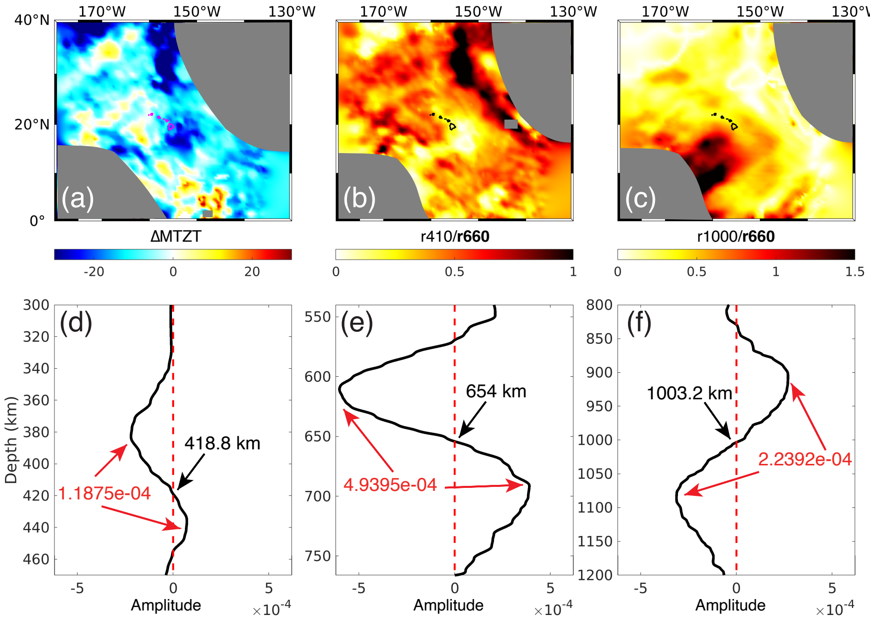

Fig. S8 MTZ thickness and reflection magnitudes from RTM images shown in Fig. 5 in the *Main Text*. (a) MTZ thickness perturbation with respect to a reference value of 250 km. (b) Relative amplitude ratio of the 410 and 660 reflectors. (c) Relative amplitude ratio of the 1000 and 660 reflectors. (d)-(f) Stacked vertical profiles of the 410, 660 and 1000 km reflector images close to Hawaii (20±2.5°N and −155±2.5°W). The arrows point to picked depths (zero-crossings) and amplitudes (averaged peak-to-trough amplitudes).

Comparison with tomography models In the last few decades, tomographic imaging has successfully imaged mantle plumes and large low-shear-velocity provinces, but it has not yielded many interpretable plume structures beneath the non-instrumented oceans [6, 7]. Fig. S9(a) shows a selection of tomographic Earth models, GLAD-M25 [5] used for our RTM imaging, SEMUCB-WM1 [8], S40RTS [9], and PRI-S05 [10] to compare with our RTM images [see also 2].

We focus our attention on the unusual reflectors that we imaged around 1000 km below the Hawaiian seamounts (Fig. 2a in the *Main Text*). Both SEMUCB-WM1 and PRI-S05 show low-velocity anomalies below 1000 km, which are interpreted to plume deflections and a viscosity jump [11]. These velocity anomalies agree with the polarity change observed from our RTM image, which indicates an impedance reversal at that depth. The low-velocity anomaly and the imaged reflector agree that the anomaly diminishes at the northwestern end of the Hawaiian seamount chain. However, the RTM image shows more topography on the discontinuity.

Fig. S9(b) shows the depth profile of globally-averaged shear wavespeed perturbations. Of note is that a transition from positive to negative perturbations occurs around 800 km depth. Three out of these four tomography models suggest extreme low shear-wave velocity anomalies between 1000 km and 1400 km depth.

Given the relative smoothness of most tomography models made with low-frequency data, we interpret the mild transition apparent in those models as the subdued signature of sharper discontinuities which, in this paper, we have imaged using the appropriate seismic phases via migration.

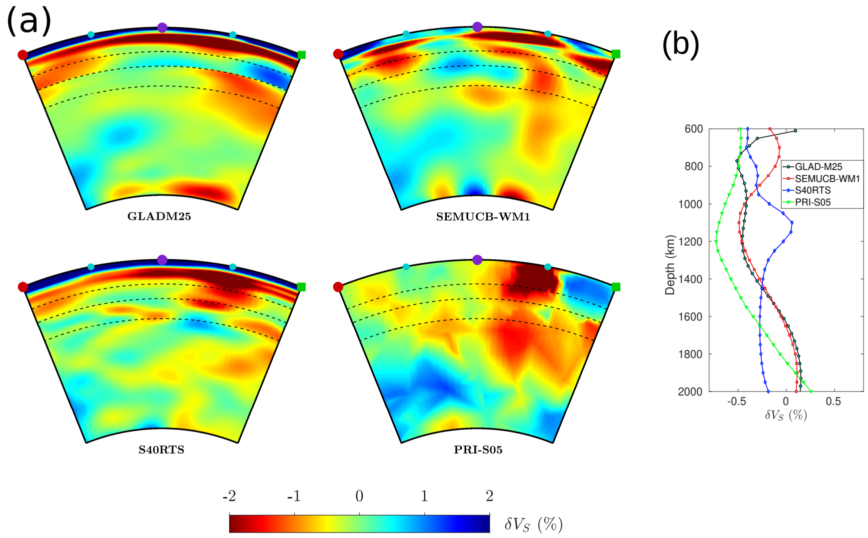

Fig. S9 Tomographic images of the mantle from published models. (a) Vertical cross-sections along the Hawaii seamount chain. (b) One-dimensional (1-D) averaged model of the vertical cross-sections. The 1000 km reflector imaged by our RTM method agrees well with the plume deflection seen in SEMUCB-WM1 [8]. The PRI-S05 model [10] also has some low-velocity anomalies below 1000 km, indicating plume deflection at that depth. GLAD-M25 [5] and S40RTS [9] do not show a similar structure. However, three out of four regionally-averaged 1-D perturbation models (b) support a low-velocity anomaly between 1000 km and 1400 km, indicating there may be a more abrupt (rheological?) change hidden at those depths, and whose character our images have brought in to a sharpened focus.

Supplementary References

[1] Dziewoński, A. M. & Anderson, D. L. Preliminary Reference Earth Model. *Phys. Earth Planet. Inter.* **25**, 297–356 (1981) .

[2] Koppers, A. A. P. *et al.* Mantle plumes and their role in earth processes. *Nat. Rev. Earth Env.* **2** (6), 382–401, doi: 10.1038/s43017–021–00168–6 (2021) .

[3] Claerbout, J. F. & Fomel, S. *Geophysical Image Estimation by Example* (lulu.com, 2014).

[4] Virieux, J. & Operto, S. An overview of full-waveform inversion in exploration geophysics. *Geophysics* **74** (6), WCC1–WCC26, doi: 10.1190/1.3238367 (2009) .

[5] Lei, W. *et al.* Global adjoint tomography—model GLAD-M25. *Geophys. J. Int.* **223** (1), 1–21, doi: 10.1093/gji/ggaa253 (2020) .

[6] Nolet, G. *et al.* Imaging the Galápagos mantle plume with an unconventional application of floating seismometers. *Sci. Rep.* **9**, 1326, doi: 10.1038/s41598–018–36835–w (2019) .

[7] Tsekhmistrenko, M., Sigloch, K., Hosseini, K. & Barruol, G. A tree of Indo-African mantle plumes imaged by seismic tomography. *Nature Geosci.* **14** (8), 612–619, doi: 10.1038/s41561–021–00762–9 (2021) .

[8] French, S. W. & Romanowicz, B. Broad plumes rooted at the base of the Earth's mantle beneath major hotspots. *Nature* **525**, 95–99, doi: 10.1038/nature14876 (2015) .

[9] Ritsema, J., Deuss, A., van Heijst, H. J. & Woodhouse, J. H. S40RTS: a degree-40 shear-velocity model for the mantle from new Rayleigh wave dispersion, teleseismic traveltime and normal-mode splitting function measurements. *Geophys. J. Int.* **184** (3), 1223–1236, doi: 10.1111/j.1365–246X.2010.04884.x (2011) .

[10] Montelli, R., Nolet, G., Dahlen, F. A. & Masters, G. A catalogue of deep mantle plumes: New results from finite-frequency tomography. *Geochem. Geophys. Geosys.* **7** (11), Q11007, doi: 10.1029/2006GC001248 (2006) .

[11] Rudolph, M. L., Lekić, V. & Lithgow-Bertelloni, C. Viscosity jump in Earth's mid-mantle. *Science* **350** (6266), 1349–1352, doi: 10.1126/science.aad1929 (2015) .
